# A head-to-head comparison of breast lesion’s conspicuity at contrast-enhanced mammography and contrast-enhanced MRI

**DOI:** 10.1007/s00330-024-11195-4

**Published:** 2024-12-03

**Authors:** Ambra Santonocito, Calogero Zarcaro, Layla Zeitouni, Francesca Ferrara, Panagiotis Kapetas, Thomas H. Helbich, Paola Clauser, Pascal A. T. Baltzer

**Affiliations:** 1 Department of Biomedical Imaging and Image-Guided Therapy, Division of General and Pediatric Radiology, General Hospital, Medical University of Vienna, Währinger Gürtel 18-20, 1090 Vienna, Austria; 2 Department of Biomedicine, Neuroscience and Advanced Diagnostic (Bi.N.D.), University Hospital “Policlinico P. Giaccone”, Via Del Vespro 129, 90127 Palermo, Italy; 3 Department of Radiology Section of Breast Imaging King Faisal Specialist Hospital and Research Center Riyadh, Riyadh, Saudi Arabia; 4 Catholic University of the Sacred Heart, Institute of Radiology, Largo A. Gemelli 8, 00168 Rome, Italy; 5 Breast Imaging Service, Department of Radiology, Memorial Sloan Kettering Cancer Center, 300 E 66th Street, New York, NY 10065 USA

**Keywords:** Breast cancer, Image quality enhancement, Sensitivity and specificity, Cancer screening

## Abstract

**Purpose:**

Lesion conspicuity, the relative enhancement of a lesion compared to surrounding tissue, is a new descriptor in the ACR BI-RADS 2022 CEM supplement. We compared lesion conspicuity in contrast-enhanced mammography (CEM) and contrast-enhanced MRI (CE-MRI) in patients with suspicious breast lesions.

**Materials and methods:**

IRB-approved retrospective study; three blinded readers rated 462 indeterminate or suspicious breast lesions in 388 patients (54.2 ± 11 years; range 30–90) who underwent CEM and CE-MRI from 2018 to 2022. Each lesion’s conspicuity was scored from 1 to 5, with 5 indicating excellent conspicuity. Visual grading characteristics (VGC) analysis and area under the curve (AUC) were used for comparison, with sub-analyses for benign and malignant lesions.

**Results:**

VGC analysis showed a significant AUC of 0.670 to 0.723 (*p* < 0.001) favouring CE-MRI. No lesion enhancement (score 1) was observed in 16.2% of CE-MRI and 44.7% of CEM. Excellent conspicuity was seen in 29.6% of CE-MRI and 11.9% of CEM. Sub-analysis showed higher conspicuity on CE-MRI for both malignant (AUC 0.665 to 0.732, *p* < 0.001) and benign lesions (AUC 0.734 to 0.798, *p* < 0.001). CE-MRI showed higher lesion conspicuity compared to CEM both for non-mass lesions (0.656) and for mass lesions 0.605.

**Conclusion:**

CE-MRI shows significantly higher conspicuity for benign and malignant breast lesions compared to CEM, especially for benign lesions. The low conspicuity of benign lesions on CEM may help reduce false positives in clinical practice.

**Key Points:**

***Question*** *Lesion conspicuity is a new descriptor for lesion enhancement according to the new CEM lexicon. Data correlating lesion conspicuity with malignancy likelihood are limited.*

***Findings*** *Lesion conspicuity is higher for contrast-enhanced-MRI than for contrast-enhanced mammography (CEM) for all lesions but significantly better for benign lesions.*

***Clinical relevance*** *The low conspicuity of benign lesions on CEM may reduce false-positive results, making it a valuable tool in breast cancer screening.*

## Introduction

Contrast-enhanced magnetic resonance imaging (CE-MRI) is considered the most sensitive method for detecting breast cancer [1]. Its sensitivity is based on the assessment of hypervascularised areas caused by tumour neo-angiogenesis by the application of a gadolinium-based contrast medium [2, 3]. Malignant lesions present a typical fast enhancement which allows their detection and characterisation with CE-MRI [4].

Contrast-enhanced mammography (CEM) is an emerging imaging technique that uses iodinated contrast media to highlight the vasculature of breast lesions [5]. CEM is performed using a dual-energy technique, which provides low and high-energy (HE) images [6].

Compared to mammography, recent publications have shown that CEM has a higher lesion detection rate, especially in dense breasts [7, 8]. Most studies have focused on the role of CEM in breast cancer staging and as a problem-solving technique, particularly in screening recalls due to inconclusive findings [9].

The ACR BI-RADS® Mammography 2022 supplement on CEM mentions lesion conspicuity as a new descriptor for lesion enhancement [10]. While visibility commonly refers to the visual manifestation of an object’s characteristics, conspicuity describes a relationship, being the degree to which the object is visually set in the background environment [11]. The detectability of a lesion, thus, depends both on its intrinsic characteristics and also on the characteristics of the surrounding structures. The CEM lexicon describes the lesion conspicuity relative to the degree of background parenchymal enhancement (BPE) as low, moderate, or high, where low conspicuity indicates a minimal difference between the lesion enhancement and the BPE and high conspicuity means a lesion enhancement far higher than BPE.

As there is no data in the scientific literature directly comparing the image quality of the two modalities in terms of lesion conspicuity, our study aims to compare the lesion conspicuity of CEM and MRI in patients with suspicious breast lesions.

## Materials and methods

This single-centre, retrospective, observational study was approved by the Institutional Review Board (IRB), and the need for written informed consent was waived. The images of consecutive patients who underwent CEM and CE-MRI between October 2018 and September 2022 were evaluated. Data were collected within a prospective study comparing the diagnostic value of CEM to CE-MRI in a problem-solving setting (Ethics Review Board number 2282/2019).

The study included women with indeterminate mammographic or ultrasound findings (BI-RADS 0, 3) and suspicious lesions (BI-RADS 4, 5). Our population was heterogeneous. From the total of 388 patients were included in the study:

-17% (*n* = 66/388) had a personal history of breast cancer that had already been operated on.
-83% (*n* = 322/388) had not received any operation for breast cancer

- 91.3% (*n* = 294/322) had undergone a screening recall or follow-up examination (of which 51.7% (*n* = 152/294) for indeterminate lesions and 48.3% (*n* = 142/294) for suspicious lesions),
- 8.7% (*n* = 28/322) presented suspicious symptoms

Exclusion criteria were CEM and CE-MRI performed more than one month apart the absence of one of the two examinations, and the lack of a reference standard.

The standard of reference was histology obtained by imaging-guided needle biopsy (core-biopsy or vacuum-assisted biopsy) or after surgery for all suspicious lesions and one-year follow-up for non-suspicious lesions.

### Imaging acquisition

#### Contrast-enhanced mammography

The system used to perform the examinations was a Mammomat Revelation unit (Siemens, Erlangen, Germany). During a single breast compression, a dual-energy examination consisting of high-energy (HE; 49 kVp) and low-energy (LE; 26–32 kVp) images was performed sequentially. An iodinated non-ionic contrast agent (Iomeron® 400, Bracco) was administered at 1 mL/kg body weight at a rate of 3 mL/s using a power injector (Ulrich Medical). Following contrast injection, 20 mL of saline was flushed. Image acquisition started 90*–*120 s after contrast injection. The examination was performed as follows: craniocaudal (CC) of the affected side, CC of the contralateral side, mediolateral oblique (MLO) of the affected side, and MLO of the contralateral side. The generation of subtracted CEM images was performed by weighted subtraction using a fully automated, locally adjusted, tissue thickness-dependent subtraction factor.

#### Breast magnetic resonance

Breast CE-MRI was performed on either 1.5-T or 3-T scanners, with dedicated breast coils and patients in the prone position. All protocols included a T2-weighted sequence and a T1-weighted series acquired before and after injection of a gadolinium-based contrast agent, in accordance with international guidelines and recommendations. A single-shot diffusion-weighted echo planar imaging (EPI) sequence (DWI) at b 0 and 800 s/mm^2^ was also included.

The scanner software automatically generated the ADC maps used for evaluation using a mono-exponential fit of the high and low b data.

### Imaging analysis

The images were independently assessed by three fellowship-trained breast radiologists with 3, 4, and 6 years of experience in breast imaging. The clinical data of the patients, the presence and location of the lesions and their histopathological findings were blinded to the readers. Evaluations were performed on dedicated workstations in separate sessions. Readers assessed the CEM images in the first reading session and the MR images in the second session, with a least 2 weeks of washout period to avoid bias due to readers recalling specific cases.

Readers evaluated on CEM the breast density on LE images, following the ACR BI-RADS 5th edition. On CEM, the lesions were described in terms of their characteristics, location, side, and size. This was done using a mammography lexicon associated with descriptors for internal enhancement characteristics (homogeneous, heterogeneous, rim) when enhancement was detected. For recombined (RC) image-only findings, the descriptors applied were mass, non-mass enhancement or enhancing asymmetry. The descriptors were assessed in both CC and MLO projections. On CE-MRI, readers assessed the amount of fibroglandular tissue on native T1-weighed sequences—with and without fat subtraction—the size and the enhancement characteristics using T1-weighted post-contrast sequences using a CE-MRI lexicon. Lesion were measured on LE and RC images on CEM, if a lesion was not visible on both the lesion size was measured as 0 by readers. Lesions were measured on T1w post-contrast sequences on CE-MRI, if the enhancing lesion was not visible, lesion size was measured as 0 by readers.

Readers assessed the lesion conspicuity in three separate categories (low, moderate, and high) as described in the CEM lexicon. Subsequently, an evaluation of lesion conspicuity was conducted utilising a five-point categorical scale based on image quality criteria, with the objective of discerning the subtlest differences in conspicuity. Each reader assessed a lesion conspicuity score for the identified lesions in each image.

This scoring system consisted of:

Grade 1—Not visible: The lesion does not show enhancement.

Grade 2—Poor conspicuity: The lesion is barely discernible, with significant difficulty in identification.

Grade 3—Fair conspicuity: The lesion is moderately visible but lacks clear distinction from surrounding tissues.

Grade 4—Good conspicuity: The lesion is clearly visible with good contrast and separation from surrounding structures.

Grade 5—Excellent conspicuity: The lesion is very well delineated, with clear and distinct visibility.

After readings, a fourth reader, who was not involved in the image analysis, matched the histopathological findings with the reading to proceed with the statistical analysis.

### Statistical analysis

Statistical analysis was performed using SPSS 27.00 (SPSS, IBM) and Med-Calc 20.216 (MedCalc Software Ltd.) software.

Categorical variables were reported as absolute numbers and percentages, and continuous variables as mean ± standard deviation (SD).

Univariate non-parametric Spearman correlation analysis was performed to identify potential covariates influencing lesion enhancement on CEM.

Data were analysed using visual grading characteristics (VGC) analysis by calculating the area under the receiver operating characteristic (ROC) curve with 95% confidence intervals (95%CI).

In VGC analysis, readers use a multi-level rating scale to indicate their evaluation of the fulfilment of specific image quality criteria [12] This can be described as an iterative image criterion scoring process in which the reader adjusts the criterion threshold, in a similar way to a reader modifying the threshold using ROC scale steps to indicate confidence in positive or negative decisions.

The classification parameter was the ordinal visual grading scale (e.g. lesion conspicuity), and the reference criterion was the image acquisition method. The study was powered to detect an AUC_VGC_ of 0.6 (zero hypothesis equal lesion conspicuity between CEM and CE-MRI indicated by an AUC_VGC_ of 0.5) at alpha and beta errors of 5% and 20%. The analysis was performed for all lesions and for malignant and benign lesions separately. Mann–Whitney test was performed to evaluate the median lesion conspicuity scores of malignant and benign lesions per reader. A further analysis was conducted on a subset of the data, focusing on lesion types, mass and non-mass lesions. Inter-operator agreement was assessed using the Fleiss’ kappa. Fleiss’ kappa coefficients were interpreted as follows: 0.21–0.40, minimal agreement; 0.41–0.60, moderate agreement; 0.61–0.80, substantial agreement; 0.81–0.90, strong agreement; > 0.90, almost perfect agreement.

## Results

Our study included 407 female patients with 483 suspicious breast lesions. Twenty-one lesions were excluded from our sample (in nineteen patients) because one of the two examinations was not available (absence of CE-MRI in ten; absence of CEM in nine) or reference standard exams were not performed (two patients with less than 12 months of follow-up).

**Fig. 1:**
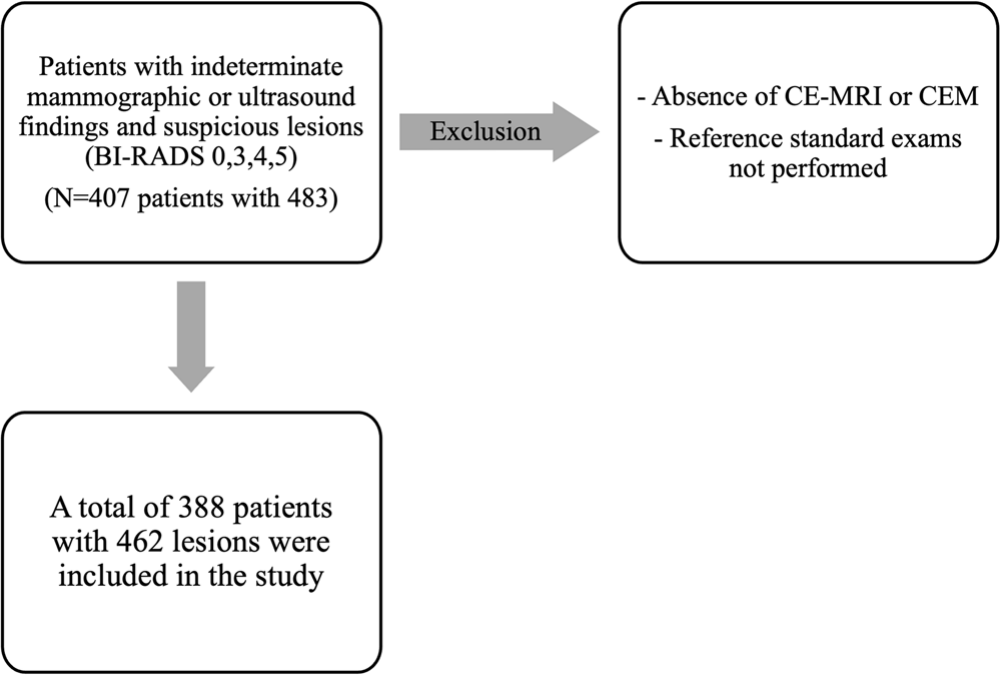
Flow chart of patient inclusion and exclusion criteria

Finally, a total of 388 patients with 462 lesions were analysed (Fig. 1). The patients had a mean age of 54.2 years (SD ± 11) with an age range between 30 and 90 years.

### Characteristics of the study population

Out of the 462 suspicious lesions analysed, 337 underwent histological verification. Of these, 160 were diagnosed as malignant (47.4%) and 177 as benign (52.6%). The remaining lesions underwent radiological follow-up.

The most frequent malignant lesion was invasive ductal carcinoma with foci of ductal carcinoma in situ (30%), while the most frequent benign lesion was fibro-adenomatous hyperplasia (19.2%). Details of the pathological analysis are shown in Table 1.

**Table 1:** Pathological analysis of breast lesions underwent histological verification

Malignant lesions	
Invasive ductal carcinoma with foci of ductal carcinoma in situ	48 (30%)
Invasive ductal carcinoma	33 (20.6%)
Ductal carcinoma in situ	44 (27.5%)
Invasive lobular carcinoma	9 (5.6%)
Others	20 (12.5%)

Benign lesions	
Fibroadenomatous hyperplasia	34 (19.2%)
Adenosis	21 (11.8%)
Fibrocystic changes	22 (12.4%)
Adipose tissue necrosis	12 (6.7%)
Intraductal papilloma	41(23.2%)
Radial scar	2 (1.1%)
Granuloma	1 (0.5%)
Others	49 (27.6%)

### Radiological characteristics of the lesions

Table 2 summarises the descriptive variables of the breast lesions.

**Table 2:** Patient and lesion characteristics

	*N*	% or SD
Age		
	54.2	11
Lesion size		
CEM	17.8	15.5
CE-MRI	18.7	18.2
Lesion side		
Right	220	47.6
Left	242	52.4
ACR breast density		
A	28	7.2
B	164	42.3
C	148	38.1
D	49	12.6
Lesion type on CEM		
No Lesion on LE images	121	26.3
Mass	105	22.7
Microcalcification	110	23.7
Asymmetry	108	23.5
Mixed	18	3.9
Enhancement type on CEM		
No lesions	180	39.0
Mass	145	31.5
Non-mass	101	21.9
Enhanced asymmetry	35	7.6
Lesion type on CE-MRI		
Mass	186	42.2%
Non-mass	143	32.4%
Foci	23	5.2%
Mixed	22	4.9%

Breast composition was found to be dense in 50.6% of patients (ACR C and D).

The average lesion size was 17.8 $\pm$ 15.5 on CEM and 18.7 $\pm$ 18.2 on CE-MRI and did not differ significantly.

Malignant lesions were predominantly assessed as masses (35.2%), while benign lesions were frequently characterised as microcalcifications (23.7%). Mass enhancement was the most common lesion type on both CEM (30.3%) and CE-MRI (42.2%).

### Visual grading and visual grading characteristics analysis

**Fig. 2:**
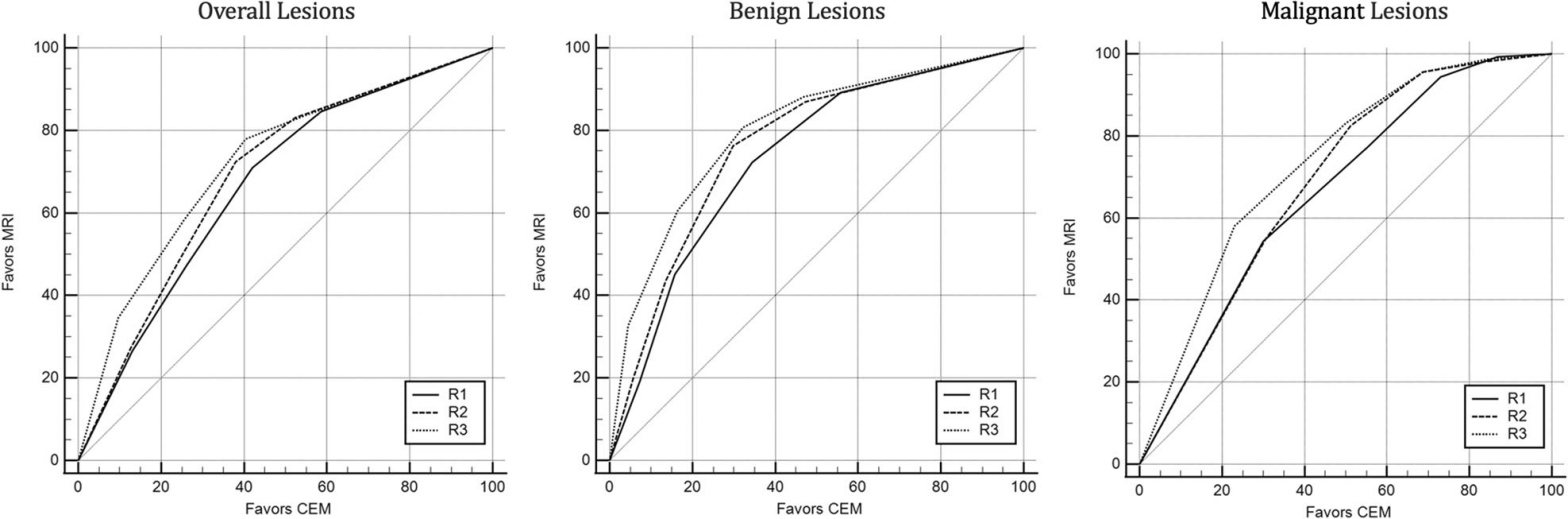
Visual grading characteristics for lesion conspicuity in overall, benign and malignant lesions

VGC analysis showed a statistically significant difference in lesion conspicuity in favour of CE-MRI for all lesions, with area under the ROC curve (AUC) ranging from 0.670 to 0.723 (*p* < 0.001) (Fig. 2). No lesion enhancement (score 1) was observed, in average, in 16.2% on CE-MRI and in 44.7% on CEM. Excellent lesion conspicuity (score 5) was observed, on average, in 29.6% of lesions on CE-MRI and in 11.9% on CEM.

The sub-analysis of malignant and benign lesions showed a statistically significant difference in lesion conspicuity in favour of CE-MRI for both lesion types. The AUC ranged from 0.665 to 0.732 (*p* < 0.001) for malignant lesions and from 0.734 to 0.798 (*p* < 0.001) for benign lesions (Fig. 2). Malignant lesions, on average, showed no lesion enhancement in 1.3% on CE-MRI and in 15% on CEM and excellent conspicuity in 56.2% on CE-MRI and in 27.9% on CEM.

Benign lesions showed, on average, no lesion enhancement in 11.7% of CE-MRI and in 49.9% of CEM and excellent conspicuity in 23.9% of CE-MRI and in 11.9% of CEM. The results of the lesion conspicuity score are summarised in Table 3, and the results of the VCG analysis are in Table 4.

**Table 3:** Lesion conspicuity 5 grade scoring system

	Overall lesions		Benign lesions		Malignant lesions	
	CEM	CE-MRI	CEM	CE-MRI	CEM	CE-MRI
Grade 1	207 (44.8%)	75 (16.2%)	89 (50.1%)	21 (11.7%)	24 (15%)	2 (1.3%)
Grade 2	69 (15%)	47 (10.2%)	31 (17.7%)	21 (11.9%)	24 (14.8%)	6 (3.5%)
Grade 3	69 (14.9%)	102 (22.2%)	30 (17.1%)	47 (26.6%)	29 (17.9%)	23 (14.2%)
Grade 4	62 (13.4%)	101 (21.9%)	16 (9.2%)	46 (26%)	39 (24.4%)	40 (25.2%)
Grade 5	55 (11.9%)	137 (29.6%)	10 (5.8%)	42 (23.9%)	45 (27.9%)	89 (55.8%)

**Table 4:** Visual grading characteristic analysis of lesion conspicuity

	R1			R2			R3		
	AUC	95%CI	*p*-value	AUC	95%CI	*p*-value	AUC	95%CI	*p*-value
General lesions	0.670	0.639–0.701	0.001	0.692	0.661–0.721	0.001	0.723	0.693–0.752	0.001
Malignant lesions	0.665	0.610–0.716	0.001	0.688	0.634–0.739	0.001	0.732	0.680–0.779	0.001
Benign lesions	0.734	0.685–0.780	0.001	0.763	0.715–0.806	0.001	0.798	0.752–0.839	0.001

**Fig. 3:**
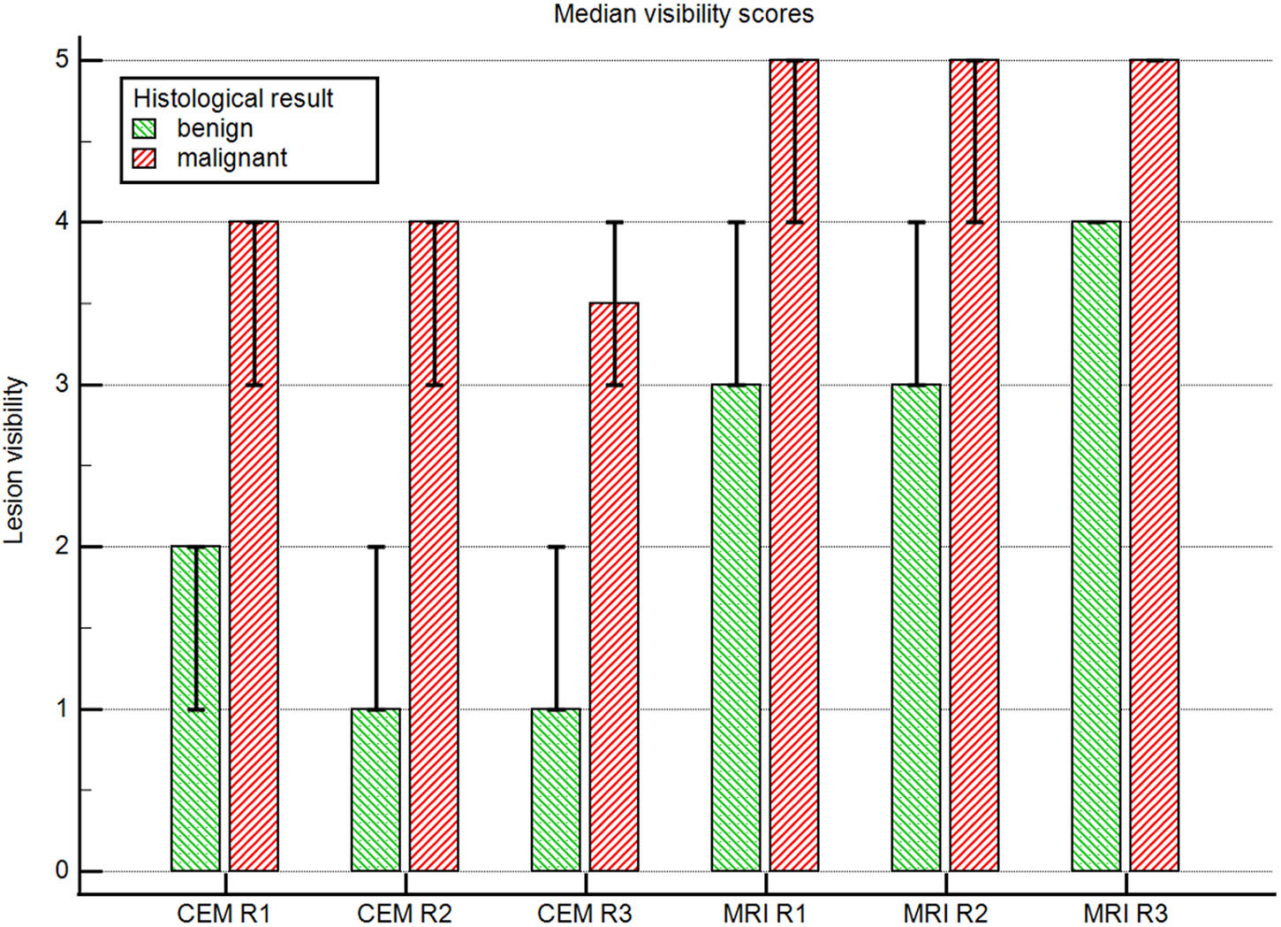
Median lesion conspicuity per reader. Mann–Whitney test

Stratifying benign and malignant lesions, the median lesion conspicuity scores showed that lesion conspicuity was higher on CE-MRI than on CEM. Notable, the difference was more pronounced for benign lesions (Fig. 3).

Analysis of further lesion types indicated a statistically significant difference in lesion conspicuity between the two imaging modalities, with CE-MRI demonstrating a higher non-mass lesion conspicuity compared to CEM for overall lesions (AUC = 0.651, *p* < 0.001), benign lesions (AUC = 0.621, *p* = 0.05), and malignant lesions (AUC of 0.656, *p* = 0.02). The difference was smaller for mass lesions (with an AUC of 0.584 for overall lesions (*p* = 0.03), an AUC of 0.580 for benign lesions (*p* = 0.2), and an AUC of 0.605 for malignant lesions (*p* = 0.17).

### Correlation between lesion conspicuity and lesion features

The Spearman correlation analysis showed a positive correlation between the average lesion conspicuity on CEM and both lesion size (*r* = 0.355, *p* < 0.001) and malignant histology (vs. benign) of enhancing lesions (*r* = 0.455, *p* < 0.001). However, no significant correlation was present for age (*r* = −0.011, *p* = 0.806) and ACR breast density (*r* = −0.025, *p* = 0.593).

### Inter-reader agreement

Assessment of inter-reader agreement for grading lesion conspicuity showed moderate results for both CE-MRI (*κ* = 0.48) and CEM (*κ* = 0.59). For malignant lesions, an inter-reader agreement was minimal for CE-MRI (*κ* = 0.37) and moderate for CEM (*κ* = 0.50) as well as for benign lesions with an agreement of 0.36 and 0.50, respectively.

## Discussion

In this intra-individual retrospective study, we compare the new CEM descriptor-defined “lesion conspicuity” in CEM and in MRI in patients with suspicious breast lesions.

**Fig. 4:**
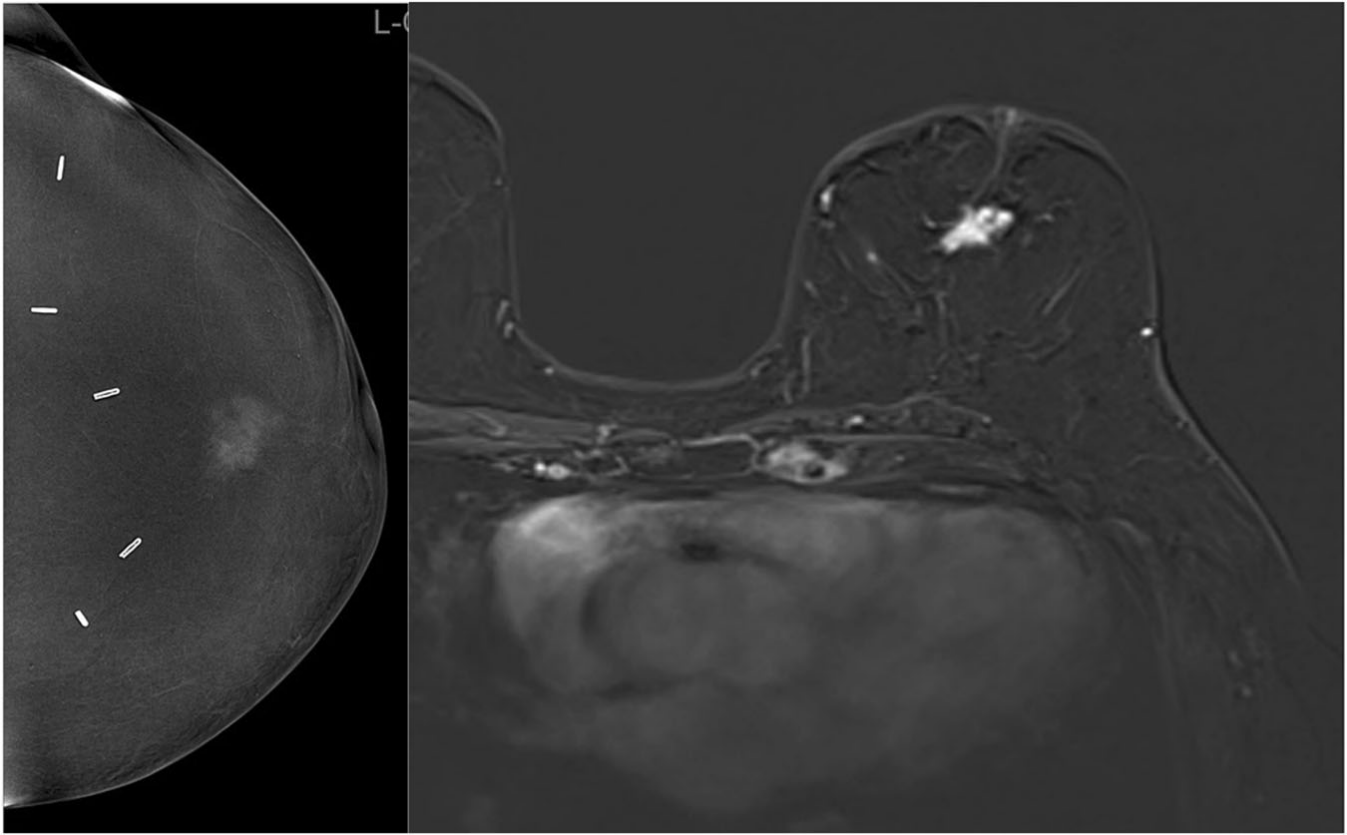
58-year-old woman with a local recurrence 8 years after the first diagnosis (triple-negative breast cancer). The lesion is clearly visible in both modalities, but conspicuity was rated higher on CE-MRI

**Fig. 5:**
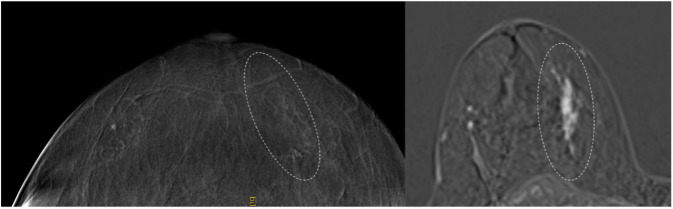
48-year-old woman with segmental pleomorphic calcifications in the medial right breast (DCIS, G3). Segmental heterogeneous non-mass enhancement is clearly visible in CE-MRI (right) with high conspicuity, but conspicuity was low in CEM (left). Note that the recombined CEM image has been mirrored to improve image comparison

Our findings indicate that CE-MRI provides higher lesion conspicuity than CEM (Figs. 4 and 5).

This finding was observed in both benign and malignant lesions, although the difference in lesion conspicuity was less evident between CEM and CE-MRI for malignant lesions. Lesion conspicuity of benign lesions was significantly better on CE-MRI. In our study, malignant lesions showed no enhancement in 1.3% of CE-MRI and in 15% of CEM, while benign lesions presented no enhancement in 11.7% of CE-MRI and in 50.1% of CEM. Also, the lesion type analysis showed higher lesion conspicuity on MRI than CEM for non-mass lesions, whereas a smaller difference between the two modalities was found for mass lesions.

The observation that CE-MRI detects lesions with higher conspicuity than CEM suggests that CE-MRI may be a more sensitive imaging modality for detecting subtle breast lesions [13–15], such as early or non-invasive breast cancer, that might be missed or misinterpreted due to low conspicuity on CEM [16]. This aspect may be attributed to the fact that MRI is a three-dimensional imaging modality with higher intrinsic contrast resolution than CEM being a two-dimensional projection imaging modality. Lack of superposition on CE-MRI may provide a better distinction between the surrounding area and the lesion itself, greatly improving lesion conspicuity.

Consistent with the conclusions of Kim et al [17] and Pötsch et al [18], our study challenges the notion that an absence of enhancement on CEM can exclude malignancy as accurately as a negative CE-MRI scan. Our data suggest the relevance of additional morphological findings in lesions with no enhancement, minimal or mild lesion conspicuity in CEM. As stated in the CEM supplement of the ACR BI-RADS and confirmed in further studies [19–21], it is important to include the interpretation of both LE and RC images when evaluating a lesion. Therefore, if a suspicious morphological finding is present on LE images with no enhancement or minimal lesion conspicuity on RC images, further investigation or needle biopsy is necessary.

Prior research has shown, that a lower lesion conspicuity on CEM is linked to their lower intrinsic biological aggressiveness [22, 23]. Such less aggressive or non-invasive cancers are often characterised by speculations and architectural distortions due to the desmoplastic reaction of the host or by calcifications and are thus easily identified on LE images [24, 25].

The observation that benign lesions show less lesion conspicuity than malignant ones is a strength for the potential future use of CEM as a breast cancer screening tool. This fact is also supported by the lower lesion conspicuity on CEM than MRI for non-mass lesions that can be assumed as benign or of low aggressiveness [26, 27]. As demonstrated by the study conducted by Grazynska et al [28], the absence of enhancement indicates the benign nature of the lesion and, in the absence of suspicious LE findings, may lead to a downgrading of BI-RADS 4 to BI-RADS 3 lesions. Consequently, the combined evaluation of findings visualised on LE images, and the degree of enhancement could reduce the risk of overdiagnosis and thus improve the accuracy of the screening process. As demonstrated in the study by Cozzi et al [29], the use of CEM in patients recalled from screening has the potential to reduce the biopsy rate by 16.4%, while maintaining high sensitivity.

The univariate analysis shows specific associations between the average enhancement of CEM and certain lesion-related features of the breast. The positive correlation between lesion size and lesion conspicuity on CEM suggests that CEM is effective in highlighting or enhancing larger lesions, making them more distinguishable in imaging. These findings are in agreement with the recent literature that showed that small lesions (< 10 mm) could be associated with a lower or a lack of enhancement in CEM [18]. Additionally, the positive correlation with the histology of enhancing lesions strengthens the evidence that CEM provides valuable information about the nature of the lesions [17, 30]. The CEM performance appears to be consistent across different age groups, as there is no significant correlation with age. Additionally, the absence of a significant correlation with ACR breast density suggests that enhancement in CEM is not influenced by breast density as categorised by the ACR scale. This finding is consistent with a recent meta-analysis by Lin et al [31], which confirmed the high diagnostic value of CEM in suspicious lesions in dense breasts and a study by Nicosia et al, which reported that lesion conspicuity performance is not affected by breast density [19].

The study also observed moderate inter-reader agreement for both techniques. These findings suggest that interpreting the conspicuity of lesions in both MRI and CEM should be approached with caution, and further investigation to enhance reliability may be required. Notably, CEM evaluations demonstrated slightly more consistent results, which underscores its simplicity in interpretation and, therefore, a potential advantage for use in both screening and diagnostic contexts.

Our study has limitations due to its retrospective, single-centre design. First, only CEM images from one vendor and one device were investigated. The results, therefore, do only apply to the specific equipment used in this study. In addition, CE-MRI images were acquired by multiple scanners, including several vendors, and both 1.5 T and 3 T with heterogeneous imaging protocols and differences in lesion conspicuity on CE-MRI may exist. However, it is well known that lesion conspicuity is not an issue in breast CE-MRI in case of sufficient image quality [32].

Additionally, we did not examine potential confounding factors such as BPE, which may mask lesions.

## Conclusion

The study shows that lesion conspicuity on CE-MRI is higher as compared to CEM, a finding more distinct in benign lesions. This does not necessarily indicate a lower diagnostic performance of CEM as the interpretation combines LE and RC images. Therefore, the results do not oppose CEM as a valuable tool for diagnosing breast lesions which is not limited to those cases only when CE-MRI is not available. The low conspicuity of benign lesions on CEM may help to reduce false positives, and the immediate availability of CEM may facilitate a one-stop approach in the assessment of breast lesions in clinical practice.

## Supplementary information

ELECTRONIC SUPPLEMENTARY MATERIAL

## Abbreviations

AUC: Area under the curve
BPE: Background parenchymal enhancement
CEM: Contrast-enhanced mammography
CE-MRI: Contrast-enhanced magnetic resonance imaging
HE: High energy
LE: Low energy
RC: Recombined image
ROC: Receiver operating characteristic
VGC: Visual grading characteristics
