# Supplementary material for: A head-to-head comparison of breast lesion’s conspicuity at contrast-enhanced mammography and contrast-enhanced MRI
Source: Eur Radiol. 2024 Dec 3;35(6):3070–9. doi: 10.1007/s00330-024-11195-4 (PMC12081499; doi:10.1007/s00330-024-11195-4)
Supplement: Supplementary file 1 — ELECTRONIC SUPPLEMENTARY MATERIAL [file 330_2024_11195_MOESM1_ESM.docx]

| Supplemental Table 1. Lesion Conspicuity according to BI-RADS supplement for CEM 2022 | | | | | | |
| --- | --- | --- | --- | --- | --- | --- |
|  | General | | Malignant | | Benign | |
|  | N | % | N | % | N | % |
| Low | 122 | 43.1 | 122 | 32.2 | 52 | 52.5 |
| Moderate | 89 | 31.4 | 89 | 32.9 | 32 | 32.3 |
| High | 72 | 25.4 | 72 | 34.9 | 15 | 38.5 |
